# New Acylglycosides Flavones from Fuzhuan Brick Tea and Simulation Analysis of Their Bioactive Effects

**DOI:** 10.3390/ijms20030494

**Published:** 2019-01-24

**Authors:** Ying Lu, Yingjie He, Shihao Zhu, Xiaohong Zhong, Dong Chen, Zhonghua Liu

**Affiliations:** 1 National Research Center of Engineering Technology for Utilization of Functional Ingredients from Botanicals, Hunan Agricultural University, Changsha 410128, China; luying960522@163.com (Y.L.); yingjiehe272@163.com (Y.H.); zsh190305763@163.com (S.Z.); 2 Horticulture and Landscape College, Hunan Agricultural University, Changsha 410128, China; xh-zhong@163.com; 3 Drinkable Plant Research Institute (Tea Research Center), Guangdong Academy of Agricultural Sciences, Guangzhou 510000, China

**Keywords:** Fuzhuan brick tea, acylglycosides flavones, camelliquercetisides, camellikaempferosides, molecular docking simulation

## Abstract

Four novel acylglycosides flavones (AGFs) including two quercetin acylglycosides and two kaempferol acylglycosides were isolated from Fuzhuan brick tea (FBT) as follows: quercetin 3-*O*-[*α*-*l*-rhamnopyranosyl (1→3)] [2-*O’’*-*(E)*-*p*-coumaroyl] [*β*-*d*-glucopyranosyl (1→3)-*α*-*l*-rhamnopyranosyl (1→6)]-*β*-*d*-galactoside was named as camelliquercetiside E (**1**), quercetin 3-*O*-[*α*-*l*-rhamnopyranosyl (1→3)] [2-*O’’*-*(E)*-*p*-coumaroyl] [*α*-*l*-rhamnopyranosyl (1→6)]-*β*-*d*-galactoside was named as camelliquercetiside F (**2**), kaempferol 3-*O*-[*α*-*l*-arabinopyranosyl (1→3)] [2-*O’’*-*(E)*-*p*-coumaroyl] [*β*-*d*-glucopyranosyl (1→3)-*α*-*l*-rhamnopyranosyl (1→6)]-*β*-*d*-glucoside was named as camellikaempferoside D (**3**), kaempferol 3-*O*-[*α*-*l*-arabinopyranosyl (1→3)] [2-*O’’*-*(E)*-*p*-coumaroyl] [*α*-*l*-rhamnopyranosyl (1→6)]-*β*-*d*-glucoside was named as camellikaempferoside E (**4**). Chemical structures of AGFs were identified by time-of-flight mass (TOF-MS) and NMR spectrometers (^1^H NMR, ^13^C NMR, ^1^H-^1^H COSY, HMBC and HSQC), and the MS^2^ fragmentation pathway of AGFs was further investigated. The inhibitory abilities of AGFs and their proposed metabolites on *α*-glucosidase and HMG-CoA reductase were analyzed by molecular docking simulation, and the results suggested that inhibitory activities of AGFs were significantly affected by acyl structure, number of glycosyl and conformation, and part of them had strong inhibitory activities on *α*-glucosidase and HMG-CoA reductase, suggesting that AGFs and their metabolites might be important ingredients that participate in the regulation of hypoglycemic and hypolipidemic effects. The results provided new AGFs and research directions for the practical study of FBT health functions in future.

## 1. Introduction

Fuzhuan brick tea (FBT), as a black tea with a large consumer base in China, it is a common health drink that is processed and fermented from the plant leaves of *Camellia sinensis* [1,2]. In recent years, a lots of chemical compounds, such as phenols, flavones, flavone glycosides, alkaloids, triterpenoids and steroids, have been separated from FBT [1,3,4,5,6], and chemical constituents with various of biological activities have been studied. To date, a series of remarkable health functions of FBT, such as antihyperlipidemia, antihyperglycemia, antiobesity, antidysentery, antibacterial, antioxidant, etc. [1,2,7,8,9], have been identified. However, due to the complexity of chemical components and the diversity of biological activities of FBT, the main molecular mechanisms of bioactivity regulations, such as main antihyperlipidemia and antihyperglycemia effects, are still unclear [10]. Therefore, while continuing the work of separation and purification of chemical compounds, the molecular mechanisms of the main health effects of FBT should also be studied at the same time.

In this study, four novel acylglycosides flavones (AGFs) were isolated from FBT by a combination of high-speed counter-current chromatography (HSCCC) and preparative high-performance liquid chromatography (prep-HPLC), and their structures were characterized by quadrupole time-of-flight mass (Q-TOF-MS) and nuclear magnetic resonance spectrometers (^1^H NMR, ^13^C NMR, ^1^H-^1^H COSY, HMBC and HSQC). Moreover, as a powerful platform for screening active ingredients, the molecular docking technique has been widely applied to screen target-specific compounds [11], and this was employed to explore hypoglycemic and hypolipidemic capabilities of the isolated AGFs and their potential metabolites in silico, by analyzing their inhibitory abilities on *α*-glucosidase and HMG-CoA reductase, respectively [12,13]. The results provide new AGFs for the further practical hypoglycemic and hypolipidemic study of FBT health functions in the future.

## 2. Results and Discussion

### 2.1. Structure Identification

**Figure 1:**
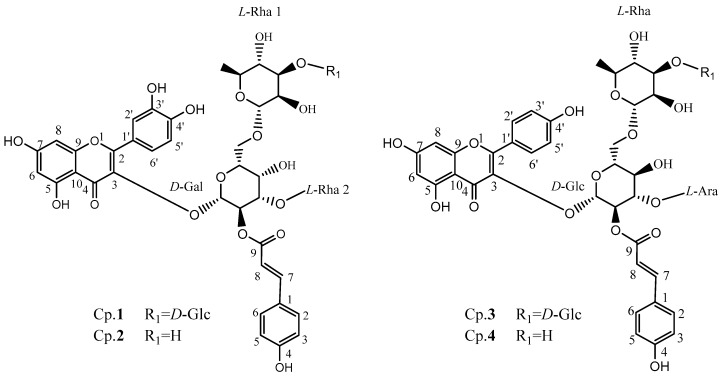
Chemical structures of acylglycosides flavones isolated from Fuzhuan brick tea.

Four of the purified compounds were identified by Q-TOF-MS and NMR, as shown in Figure 1, and the detailed NMR data of those compounds are described in Table 1. The original data of isolated compounds could be found in the Supplementary Materials (Figure S1). Briefly, all of them can be depicted as followed.

Compound **1**: The molecular formula was determined to be C_48_H_56_O_27_ based on its [M+H]^+^ ion at *m*/*z* 1065.3085 by a Q-TOF-MS analysis. The ^1^H NMR and ^1^H-^1^H COSY data revealed the presence of a quercetin moiety: δ_H_ 7.57 (1H, m), 6.89 (1H, d, *J* = 8.4 Hz) and 6.82 (1H, s) for the 3’,4’-disubstitution on the B-ring, and *δ*_H_ 6.19 (1H, d, *J* = 4.8) and 6.40 (1H, d, *J* = 4.8) for the 5,7-disubstitution on the A-ring; a *p*-coumaric acid moiety: *δ*_H_ 6.37 (1H, t, *J* = 4.2), 6.83 (2H, s, *J* = 4.2), 7.47 (2H, dd, *J* = 8.4) and 7.72 (1H, m); four saccharides (four anomeric protons) were observed: *δ*_H_ 5.56 (1H, d, *J* = 7.8), 4.63 (1H, s), 4.61 (1H, d, *J* = 7.8) and 4.60 (1H, s) respectively. The ^13^C NMR and HSQC experiments also suggested the presence of four saccharides, as four anomeric carbons were observed at δ_C_ 103.9, 114.8, 113.8 and 104.2, respectively. Acid hydrolysis was performed, and four monosaccharides were detected and identified as one *d*-glucose, one *d*-galactose and two *L*-rhamnoses for this compound from HPLC analysis by using (*S*)-(−)-1-phenylethylamine as the chiral-derivatization reagent. The configurations of the anomeric carbons of the galactose, the glucose and two rhamnoses were defined as *β*-d, *β*-d and *α*-*l*, respectively, based on their corresponding proton coupling constants 7.8, 7.8, 0 and 0 Hz, respectively. The glycosidic linkages were determined by the HMBC spectra as follows: (1→3) linked for Gal to quercetin as HMBC signal of *δ*_H_ 5.56 (H-1, Gal) to δ_C_ 133.6 (C-3 of quercetin moiety) was observed, (1→3) linked for Rha2 to Gal as HMBC signal of *δ*_H_ 4.60 (H-1, Rha2) to *δ*_C_ 76.1 (C-3, Gal) was observed, (1→6) linked for Rha1 to Gal as HMBC signal of *δ*_H_ 4.63 (H-1, Rha1) to *δ*_C_ 65.8 (C-6, Gal) was obtained, (1→3) linked for Glu to Rha1 as HMBC signal of *δ*_H_ 4.61 (H-1, Glu) to *δ*_C_ 98.5 (C-3, Rha1) was obtained, and (2→9) linked for Gal to coumaroyl group as HMBC signal of *δ*_H_ 5.24 (H-2, Gal) to *δ*_C_ 167.3 (C-9, coumaroyl group) was observed. Therefore, it was identified as quercetin 3-*O*-[*α*-*l*-rhamnopyranosyl (1→3)] [2-*O’’*-*(E)*-*p*-coumaroyl] [*β*-*d*-glucopyranosyl (1→3)-*α*-*l*-rhamnopyranosyl (1→6)]-*β*-*d*-galactoside, named camelliquercetiside E according to the reported data [14].

Compound **2**: The molecular formula was determined to be C_42_H_46_O_22_ based on its [M+H]^+^ ion at *m*/*z* 903.2555 by a Q-TOF-MS analysis. The ^1^H NMR and ^1^H-^1^H COSY data revealed the presence of a quercetin moiety: *δ*_H_ 6.82 (1H, d, *J* = 4.2), 7.59 (1H, m) and 6.89 (1H, d, *J* = 8.4) for the 3’,4’-disubstitution on the B-ring, and *δ*_H_ 6.19 (1H, d, *J* = 6.6) and 6.40 (1H, t, *J* = 9.0) for the 5,7-disubstitution on the A-ring; a *p*-coumaric acid moiety: *δ*_H_ 6.36 (1H, d, *J* = 12.0), 7.71 (1H, m), 7.47 (2H, dd, *J* = 3.6) and 6.83 (2H, dd, *J* = 4.2); three saccharides (three anomeric protons) were observed: *δ*_H_ 5.57 (1H, d, *J* = 8.4), 4.59 (1H, s) and 4.57 (1H, s), respectively. The ^13^C NMR and HSQC experiments also suggested the presence of three saccharides, as three anomeric carbons were observed at δ_C_ 104.0, 114.8 and 113.8, respectively. Acid hydrolysis was performed, and two monosaccharides were detected and identified as galactose and rhamnoses for this compound from HPLC analysis by using (*S*)-(−)-1-phenylethylamine as the chiral-derivatization reagent. Integrated interpretation based on ^1^H-^1^H COSY, HSQC and HMBC reveals the existence of one *d*-galactose and two *L*-rhamnoses for this compound. The configurations of the anomeric carbons of the galactose and two rhamnoses were defined as *β*-*d* and *α*-*l*, respectively, based on their corresponding proton coupling constants 8.4, 0 and 0 Hz, respectively. The glycosidic linkages were determined by the HMBC spectra as follows: (1→3) linked for Gal to quercetin as HMBC signal of *δ*_H_ 5.57 (H-1, Gal) to *δ*_C_ 133.5 (C-3 of quercetin moiety) was observed, (1→3) linked for Rha2 to Gal as HMBC signal of *δ*_H_ 4.57 (H-1, Rha2) to *δ*_C_ 76.0 (C-3, Gal) was observed, (1→6) linked for Rha1 to Gal as HMBC signal of *δ*_H_ 4.59 (H-1, Rha1) to *δ*_C_ 68.4 (C-6, Gal) was obtained, and (2→9) linked for Gal to coumaroyl group as HMBC signal of *δ*_H_ 5.24 (H-2, Gal) to *δ*_C_ 167.3 (C-9, coumaroyl group) was observed. Therefore, it was identified as quercetin 3-*O*-[*α*-*l*-rhamnopyranosyl (1→3)] [2-*O’’*-*(E)*-*p*-coumaroyl] [*α*-*l*-rhamnopyranosyl (1→6)]-*β*-*d*-galactoside, named camelliquercetiside F according to the reported data [14].

Compound **3**: The molecular formula was determined to be C_47_H_54_O_26_ based on its [M+H]^+^ ion at *m*/*z* 1035.2976 by a Q-TOF-MS analysis. The ^1^H NMR and ^1^H-^1^H COSY data revealed the presence of a kaempferol moiety: *δ*_H_ 7.02 (2H, d, *J* = 9.0) and 8.09 (2H, d, *J* = 8.4) for the 4’-monosubstitution on the B-ring, *δ*_H_ 6.40 (1H, d, *J* = 16.2) and 6.25 (1H, d, *J* = 1.8) for the 5,7-disubstitution on the A-ring; a *p*-coumaric acid moiety: *δ*_H_ 6.49 (1H, d, *J* = 1.8), 6.91 (2H, d, *J* = 9.6), 7.74 (1H, d, *J* = 15.6) and 7.56 (2H, d, *J* = 9.0); and four saccharides (four anomeric protons) were observed: 5.77 (1H, d, *J* = 7.8), 4.62 (1H, s), 4.52 (1H, d, *J* = 7.8) and 4.39 (1H, d, *J* = 6.6), respectively. The ^13^C NMR and HSQC experiments also suggested the presence of four saccharides as four anomeric carbons were observed at δ_C_ 99.3, 104.2, 101.1 and 104.1 respectively. Acid hydrolysis was performed, and three monosaccharides were detected and identified as two *D*-glucose, one *L*-rhamnose and one *L*-arabinose from HPLC analysis by using (*S*)-(−)-1-phenylethylamine as the chiral-derivatization reagent. The configurations of the anomeric carbons of the two glucoses, one arabinose and one rhamnose were defined as *β*-*d*, *α*-*l*, and *α*-*l*, respectively, based on their corresponding proton coupling constants 7.8, 7.8, 6.6 and 0 Hz respectively. The glycosidic linkages were determined by the HMBC spectra as follows: (1→3) linked for Glu to kaempferol as HMBC signal of *δ*_H_ 5.77 (H-1, Glu1) to *δ*_C_ 133.3 (C-3 of kaempferol moiety) was observed, (1→6) linked for Rha to Glu as HMBC signal of *δ*_H_ 4.39 (H-1, Rha) to *δ*_C_ 66.1 (C-6, Glu) was observed, (1→3) linked for Glu (R1)to Rha as HMBC signal of *δ*_H_ 4.52 (H-1, Glu) to *δ*_C_ 84.1 (C-3, Rha) was obtained, (1→3) linked for Ara to Glu (R1) as HMBC signal of *δ*_H_ 4.62(H-1, Ara) to *δ*_C_ 74.4 (C-3, Glu) was observed, and (2→9) linked for Glu to coumaroyl group as HMBC signal of *δ*_H_ 5.17 (H-2, Glu) to *δ*_C_ 166.5 (C-9, coumaroyl group) was observed. Therefore, it was identified as kaempferol 3-*O*-[*α*-*l*-arabinopyranosyl (1→3)] [2-*O’’*-*(E)*-*p*-coumaroyl] [*β*-*d*-glucopyranosyl (1→3)-*α* -*l*-rhamnopyranosyl (1→6)]-*β*-*d*-glucoside, named camellikaempferoside D according to the reported data [5,15,16].

Compound **4**: The molecular formula was determined to be C_41_H_44_O_21_ based on its [M+H]^+^ ion at *m*/*z* 873.2446 by a Q-TOF-MS analysis. The ^1^H NMR and ^1^H-^1^H COSY data revealed the presence of a kaempferol moiety: *δ*_H_ 6.87 (2H, d, *J* = 8.4) and 7.91 (2H, d, *J* = 8.4) for the 4’-monosubstitution on the B-ring, *δ*_H_ 6.11 (1H, s) and 5.93 (1H, s) for the 5,7-disubstitution on the A-ring; a *p*-coumaric acid moiety: *δ*_H_ 6.35 (1H, d, *J* = 15.6), 7.57 (1H, d, *J* = 15.6), 7.52 (2H, d, *J* = 7.8) and 6.79 (2H, d, *J* = 14.4); and three saccharides (three anomeric protons) were observed: 5.62 (1H, d, *J* = 7.8), 4.39 (1H, s) and 4.35 (1H, d, *J* = 5.4), respectively. The ^13^C NMR and HSQC experiments also suggested the presence of three saccharides, as three anomeric carbons were observed at δ_C_ 101.4, 114.6 and 114.6, respectively. Acid hydrolysis and chiral-derivatization, were also performed, followed by HPLC analysis, and the presence of *d*-glucose, *l*-rhamnose and *l*-arabinose was confirmed. The configurations of the anomeric carbons of the glucoses, arabinose, and rhamnose were also identified as *β*-*d*, *α*-*l*, and *α*-*l* respectively based on their corresponding proton coupling constants 7.8, 5.4 and 0 Hz, respectively. The glycosidic linkages were determined by the HMBC spectra as follows: (1→3) linked for Glu to kaempferol as HMBC signal of *δ*_H_ 5.62 (H-1, Glu) to *δ*_C_ 132.6 (C-3 of kaempferol moiety) was observed, (1→6) linked for Rha to Glu as HMBC signal of *δ*_H_ 4.39 (H-1, Rha) to *δ*_C_ 68.9 (C-6, Glu) was observed, (1→3) linked for Ara to Glu as HMBC signal of *δ*_H_ 4.35 (H-1, Ara) to *δ*_C_ 77.7 (C-3, Glu) was observed, and (2→9) linked for Glu to coumaroyl group as HMBC signal of *δ*_H_ 4.98 (H-2, Glu) to *δ*_C_ 166.1 (C-9, coumaroyl group) was observed. Therefore it can be identified as kaempferol 3-*O*-[*α*-*l*-arabinopyranosyl (1→3)] [2-*O’’*-*(E)*-*p*-coumaroyl] [*α-l*-rhamnopyranosyl (1→6)]-*β-d*-glucoside, named camellikaempferoside E according to the reported data [5,15,16].

### 2.2. Proposed MS^2^ Fragmentation Pathway of Acylglycosides Flavones

**Figure 2:**
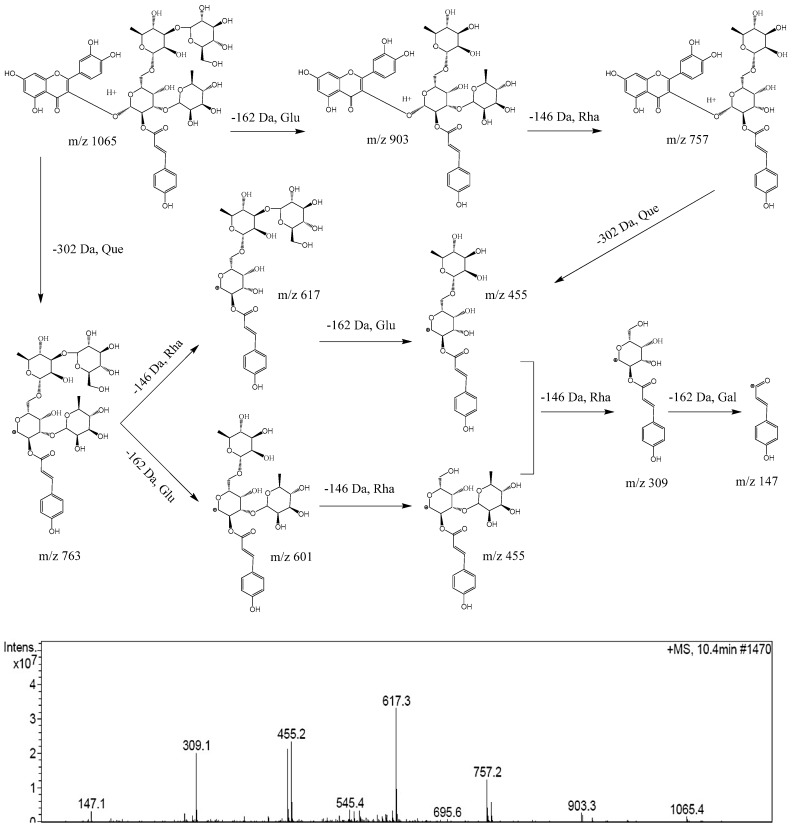
Proposed fragmentation pathway of camelliquercetiside E in positive ion mode.

The MS^2^ fragmentation pathway of camelliquercetiside E, a new acylglycosides flavone, was tentatively proposed in positive mode as shown in Figure 2. There were two fragmentation pathways. Route 1: firstly, the quasi-molecular ion at *m*/*z* 1065 was obtained, fragment at *m*/*z* 903 and fragment at *m*/*z* 757 indicated the loss of 162 Da (−Glu) and 146 Da (−Rha); then, fragment ion at *m*/*z* 455 indicated a bond rupture between the parent nucleus and glycosides with loss of 302 Da (−Que); finally, the generated acylglycosides was sequentially split into acyl ion at *m*/*z* 147 by loss of 146 Da (−Rha) and 162 Da (−Gal). Route 2: the parent nucleus (302 Da, −Que) was firstly split from the quasi-molecular ion at *m*/*z* 1065, and a new acylglycosides ion at *m*/*z* 763 was generated; then, acylglycosides were split into acyl ion at m/z 147 by loss of glycosyls sequentially at 146 Da (−Rha), 162 Da (−Glu), 146 Da (−Rha) and 162 Da (−Gal). The typical fragmentation pathways of other acylglycosides flavones were consistent with camelliquercetiside E.

### 2.3. Simulation Analysis of Potential Hypoglycemic and Hypolipidemic Effects

α-glucosidase catalyzes the final steps in the digestive process of carbohydrates, and its inhibitors (such as acarbose) can delay the absorption of carbohydrates and thus suppress postprandial blood glucose and insulin levels, which have been widely used in the treatment of patients with type 2 diabetes mellitus (T2DM) [17,18]. High blood lipid is mainly related to the density of cholesterol and low-density lipoprotein in the blood, inhibitors such as mevastatin can inhibit the HMG-CoA reductase and regulate lipid concentration [13]. Therefore, screening of the active ingredients with good inhibitory abilities on α-glucosidase and HMG-CoA reductase could better explain the proved hypoglycemic and hypolipidemic effects of FBT.

**Figure 3:**
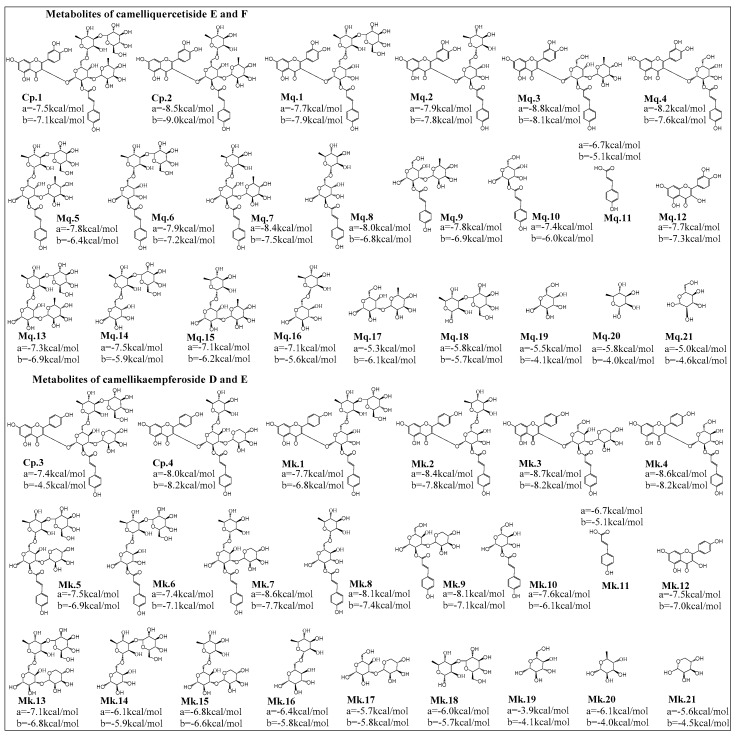
Proposed hydrolysis metabolites of acylglycosides flavones and docking score analysis of their inhibitory abilities on *α*-glucosidase (**a**) and HMG-CoA reductase (**b**). Among camelliquercetisides, metabolites Mq.**3** and prototype Cp.**2** (camelliquercetiside F) exhibited excellent inhibitory activities on α-glucosidase and HMG-CoA reductase at −8.8 kcal/mol and −9.0 kcal/mol, respectively. For camellikaempferosides, metabolites Mk.3 exhibited good inhibitory activities on α-glucosidase at −8.7 kcal/mol, Cp.**4**, Mk.**3** and Mk.**4** were calculated as the good inhibitors on HMG-CoA reductase.

Flavonoids are usually hydrolyzed into metabolites in the gastrointestinal tract and then absorbed and utilized in vivo [19], bioactive compounds might be the prototype compounds or their metabolites. Therefore, inhibitory abilities of AGFs and their proposed main metabolites on α-glucosidase and HMG-CoA reductase were calculated and analyzed by molecular docking simulation, as shown in Figure 3.

**Figure 4:**
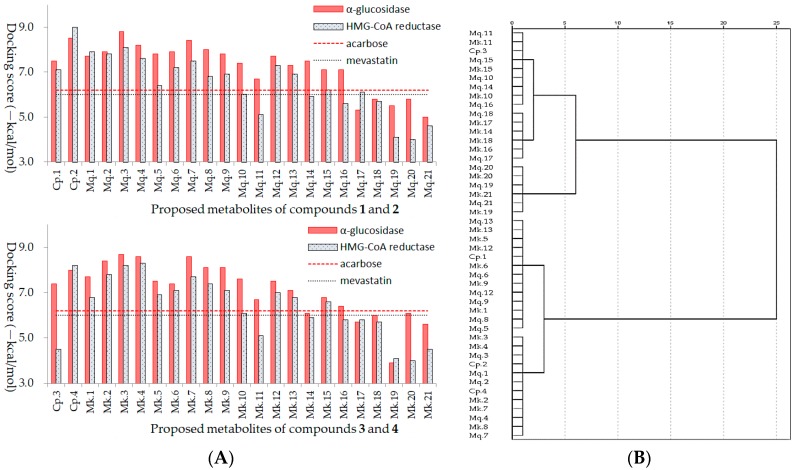
Trends analysis of metabolite inhibitory activities of acylglycosides flavones on *α*-glucosidase and HMG-CoA reductase (**A**), and clustering analysis of the homogeneity of the binding conformations (**B**).

As shown in Figure 4A, the docking scores of positive drugs acarbose and mevastatin were −6.2 kcal/mol and −6.0 kcal/mol, respectively, docking scores lower than positive drug suggest higher inhibitory ability on enzyme. In addition, clustering analysis was employed to evaluate the homogeneity of the binding conformations (Figure 4B), combined with Figure 3 and Figure 4, the results suggested inhibitory activities of AGFs were significantly affected by acyl structure and number of glycosyl. Acyl groups had a significant effect on the stability and bioactivity of flavonoids, compared with the metabolites with acyl group, the metabolites without acyl group commonly had lower inhibitory abilities, the reason might be that the acyl group structure participates in the formation of stable hydrophobic force and improve the biological activity of bioactive compound. The number of glycosyl could affect generation of steric hindrance and hydrogen bonds, lots of glycosyl groups on flavonoids lead to steric hindrance and difficult binding within the active pocket of enzymes, so the appropriate glycosyl groups and positional conformation could improve inhibitory abilities with low docking scores, which might be potential target inhibitors.

### 2.4. Molecular Mechanisms of Inhibitors on α-Glucosidase and HMG-CoA Reductase

To investigate molecular mechanisms of good inhibitors conjugated with receptors, PyMOL was employed to analyze the hydrogen bonds and hydrophobic interactions between inhibitors and amino acid residues in binding sites. As shown in Table 2, the results showed that hydrophobic forces and hydrogen bonds formed by each inhibitor with the residues of the active sites were generally consistent with the original inhibitors. The good inhibitory capacities of these compounds might be due to the formation of hydrophobic interaction with more non-polar amino acid residues and stable hydrogen bonds.

## 3. Materials and Methods

### 3.1. Chemicals and Materials

FBT was collected from XiangYi Special FBT; it was produced by Hunan Yiyang Tea Company and stored in a dry environment. Ethanol, petroleum ether, *n*-butanol, ethyl acetate, methanol and acetic acid were purchased from Sinopharm Chemical Reagent Co., Ltd. (Shanghai, China). Acetonitrile of LC-MS grade was purchased from Merck (Darmstadt, Germany). Deionized water was produced by a Milli-Q water purification system (Millipore, Billerica, MA, USA). 

### 3.2. Preparation of Crude Extract

2.0 kg powder of Fu brick tea was extracted with 16 L 95% ethanol solution by ultrasonic extraction for 1 h in triplicate. The extracts were combined and concentrated on a rotary evaporator at 55 °C to remove ethanol completely, 500 mL water was added to the concentrated extract, and then, 500 mL petroleum ether was added to the suspensions each time to extract non-target compounds until petroleum ether was colorless. Finally, aqueous layer was for further concentration and freeze-drying, and 103 g dry powder were yielded for the next separation.

### 3.3. HSCCC Separation

A TBE-300A High-speed counter current chromatography (HSCCC) (Shanghai Tauto Biotechnique Company, Shanghai, China) with three multilayer coil separation columns (i.d. 1.6 mm, volume 280 mL) was employed for the initial separation. The two-phase solvent system was composed of *n*-butanol, ethyl acetate, acetonitrile and 0.5% acetic acid (12:2:3:15, *V/V/V/V*). The upper layer was used as the stationary phase, and the lower layer was used as the mobile phase. The flow rate was 2.0 mL/min, the revolution speed was 850 rpm, the separation temperature was 25 °C, detector wavelength was 280 nm, and six peak flows were collected successively according to the chromatogram. Each fraction was concentrated and freeze-dried, yielded 20.2 g (fraction 1), 5.3 g (fraction 2), 15.5 g (fraction 3), 4.6 g (fraction 4), 5.2 g (fraction 5), 3.1 g (fraction 6) powder respectively for further purification.

### 3.4. Preparative HPLC Purification

The preparative HPLC was equipped a LC-8A system (Shimadzu Corporation, Tokyo, Japan) with Unitary C_18_ column (250 mm × 10 mm, 5μm). The mobile phase consisted of 0.5% acetic acid water (A) and methanol (B). Elution procedure was performed with a linear gradient as follows: 0→80 min, 15%→60% B. The flow rate was 5.0 mL/min, injection volume was 1.0 mL (0.5 mg each of fraction powder dissolved in methanol). The effluents were collected respectively according to the detection of HPLC peaks with multiple times, and then, each effluent was concentrated and freeze-dried into powder. Based on continuous separation procedures of HSCCC and prep-HPLC, a series of new flavones were purified. Two quercetin acylglycosides, compounds **1** (40.4 mg, 98.5%) and **2** (15.6 mg, 95.7%), were obtained from fraction 2 and fraction 3, respectively. In addition, two kaempferol acylglycosides, compounds **3** (32.8 mg, 92.6%) and **4** (27.5 mg, 97.8%), were obtained from fraction 2 and fraction 5, respectively. 

### 3.5. HPLC-Q-TOF-MS Analysis

The HPLC was equipped analytical LC20 AT-VP Plus (Shimadzu Corporation) combined with a SPD-M20A VP UV detector and a Wondasil^TM^ C_18_ column (250 mm × 4.6 mm, 5 μm). The mobile phase consisted of 0.2% formic acid water (A) and acetonitrile (B) with a linear gradient as follows: 0→60 min, 5%→30% B. The follow rate was set at 1.0 mL/min, and the detected wavelength was set at 280 nm. The purity of each powder was detected by analytical HPLC for further structural identification.

Mass spectrometry experiment was performed using a 6530 Q-TOF-MS accurate-mass spectrometer (Agilent Technologies, Palo Alto, CA, USA), operating in negative and positive electrospray ionization mode [20]. TOF data was collected from *m*/*z* 100 to *m*/*z* 1700 in centroid mode. The conditions of the Q-TOF-MS were optimized as follows: gas temperature, 310 °C; drying gas flow rate, 12 L/min; fragmentor voltage, 135 V; sheath gas temperature, 350 °C; sheath gas flow rate, 11 L/min; nebulizer gas pressure, 45 psi; capillary voltage, 3500 V; skimmer voltage, 65 V; OCT1 RF Vpp, 750 V. Identification of compounds was carried out by comparing retention time, m/z of quasi-molecular ions and MS^2^ fragmentation patterns and the literature data. Fragment ions of acylglycosides flavones were obtained in positive ion mode at collision energy of 0 ev. 

### 3.6. NMR Analysis

A Bruker 600M and 150M NMR spectrometers were employed for the identification of those isolated compounds with ^1^H NMR and ^13^C NMR. The TMS was used as the internal standard in DMSO-d6.

### 3.7. Molecular Docking

The molecular docking analysis was carried out by using *α*-glucosidase (ID: 3W37) [12] and HMG-CoA reductase (ID: 1HW8) [13] respectively through searching the protein data bank (PDB), the 3D structure of isolated compounds and their metabolites were prepared as the ligands. The active pockets of the *α*-glucosidase and HMG-CoA reductase were concentrated on the binding sites (40 × 40 × 40 Å^3^, 0.375 Å, central coordinates x = 0.301, y = −1.798, and z = −23.212) and (40 × 40 × 40 Å^3^, 0.375 Å, central coordinates x = 19.133, y = −22.431, and z = 15.679) to dock with these compounds respectively. Then the docking processes were performed according to our previous study [21,22], docking scores were of the indicator of inhibitory capacities and were calculated in triplicate.

## 4. Conclusions

In the present study, four new AGFs were successful isolated from FBT for the first time, hypoglycemic and hypolipidemic capabilities of AGFs and their metabolites were preliminarily analyzed by molecular docking simulation, some of them being identified as good α-glucosidase and HMG-CoA reductase inhibitors. The results enriched the knowledge of the AGFs and provided research direction of FBT health functions.

## Figures and Tables

**Table 1:** ^1^H and ^13^C NMR spectroscopic data of four acylglycosides flavones (600 and 150 MHz, DMSO-d_6_).

No	Cp.1		Cp.2		Cp.3		Cp.4	
	*δ*_H_ (*J* in Hz)	*δ*_C_	*δ*_H_ (*J* in Hz)	*δ*_C_	*δ*_H_ (*J* in Hz)	*δ*_C_	*δ*_H_ (*J* in Hz)	*δ*_C_
	***Quercetin***		***Quercetin***		***Kaempferol***		***Kaempferol***	
2	-	157.0	-	157.0	-	157.1	-	156.2
3	-	133.6	-	133.5	-	133.3	-	132.6
4	-	177.7	-	177.6	-	177.7	-	178.1
5	-	161.7	-	161.7	-	161.9	-	161.4
6	6.19, d (4.8)	99.6	6.19, d (6.6)	99.4	6.25, d (1.8)	98.6	5.93, s	97.9
7		167.3	-	167.2	-	164.1		164.4
8	6.40, d (4.8)	93.5	6.40, t (9.0)	93.4	6.40, d (16.2)	93.8	6.11, s	92.6
9	-	159.9	-	159.9	-	159.9	-	160.6
10	-	100.9	-	101.0	-	104.8	-	103.1
1’	-	122.1	-	122.1	-	121.7	-	125.5
2’	6.82, s	115.4	6.82, d (4.2)	116.0	8.09, d (8.4)	131.2	7.91, d (8.4)	130.7
3’	-	145.9	-	145.9	7.02, d (9.0)	115.2	6.87, d (8.4)	115.6
4’	-	148.3	-	146.7	-	159.8	-	160.6
5’	6.89, d (8.4)	117.0	6.89, d (8.4)	116.8	7.02, d (9.0)	115.2	6.87, d (8.4)	115.6
6’	7.57, m	121.8	7.59, m	121.5	8.09, d (8.4)	131.2	7.91, d (8.4)	130.7
	***p*-coumaric acid**		***p*-coumaric acid**		***p*-coumaric acid**		***p*-coumaric acid**	
9	-	167.3	-	167.3	-	166.5	-	166.1
8	6.37, t (4.2)	116.1	6.36, d (12.0)	115.9	6.49, d (1.8)	114.5	6.35, d (15.6)	116.3
7	7.72, (m)	144.5	7.71, m	145.6	7.74, d (15.6)	145.4	7.57, d (15.6)	145.4
1	-	125.9	-	125.9	-	126.1	-	130.1
2/6	7.47, dd (8.4)	129.9	7.47, dd (3.6)	129.9	7.56, d (9.0)	130.2	7.52, d (7.8)	131.1
3/5	6.83, s (4.2)	115.4	6.83, dd (4.2)	115.4	6.91, d (9.6)	115.8	6.79, d (14.4)	115.6
4	-	157.5	-	157.4	-	157.6	-	160.5
	***β-d-Gal***		***β-d-Gal***		***β-d-Glu***		***β-d-Glu***	
1	5.56, d (7.8)	103.9	5.57, d (8.4)	104.0	5.77, d (7.8)	99.3	5.62, d (7.8)	101.4
2	5.24, t (9.0)	70.8	5.24, t (9.0)	70.7	5.17, t (9.6)	70.2	4.98, t (9.0)	71.0
3	4.00	76.1	3.99	76.0	3.96	66.0	3.89	77.7
4	3.88	67.1	3.92	70.5	3.89	76.3	3.78	69.0
5	3.87	81.7	3.80	83.0	3.89	76.4	3.80	80.7
6	3.63, 3.61	65.8	3.61, 3.50	68.4	3.56, 3.41	66.1	3.73, 3.63	68.9
	***α-l-Rha 1***		***α-l-Rha 1***		***α-l-Rha***		***α-l-Rha***	
1	4.63, s	114.8	4.59, s	114.8	4.62, s	104.2	4.39, s	114.6
2	3.90	68.8	3.89	73.1	3.96	68.8	3.85	72.6
3	3.63	98.5	3.63	70.8	3.43	84.1	3.72	70.8
4	3.62	69.5	3.62	72.5	3.84	69.7	3.38	72.3
5	3.64	74.1	3.63	75.6	3.66	72.9	3.76	73.3
6	1.30, t (8.4)	16.7	1.30, t (6.6)	17.0	1.30, s	17.2	1.24, s	18.2
	***R1 (β-d-Glu)***		-		***R1 (β-d-Glu)***		-	
1	4.61, d (7.8)	113.8	-	-	4.52, d (7.8)	101.1	-	-
2	3.92	73.2	-	-	3.84	72.6	-	-
3	3.78	75.5	-	-	3.68	74.4	-	-
4	3.74	68.8	-	-	3.58	67.9	-	-
5	3.63	82.9	-	-	3.57	82.5	-	-
6	3.62, 3.61	60.7	-	-	3.51,3.50	56.1	-	-
	***α-l-Rha 2***		***α-l-Rha 2***		***α-l-Ara***		***α-l-Ara***	
1	4.60, s	104.2	4.57, s	113.8	4.39, d (6.6)	104.1	4.35, d (5.4)	114.6
2	3.90	72.5	3.90	74.8	3.82	71.1	3.84	72.3
3	3.80	69.9	3.69	70.7	3.71	75.4	3.77	75.7
4	3.61	71.2	3.62	72.5	3.68	66.8	3.75	70.7
5	3.62	73.2	3.64	74.8	3.82, 3.60	61.5	3.84, 3.63	67.2
6	1.30, t (8.4)	16.7	1.30, t (6.6)	17.0	-	-	-	-

**Table 2:** Analysis of the conformation of good inhibitors within the binding sites of the receptors.

Receptor	Compound	Score (kcal/mol)	Common Hydrophobic Residues (within a Range of 4 Å)	Number of H-Bonds	Hydrogen Bond Residues ^c^
*α*-glucosidase	acarbose ^a^	−6.2	ILE-358, ILE-396, TRP-467, TRP-432, MET-470, ILE-233, ALA-234, TRP-329, TRP-565, PHE-236, PHE-601	5	/
	Mq.3 ^b^	−8.8	ILE-396, TRP-467, TRP-432, MET-470, ILE-233, ALA-234, TRP-329, PHE-236, PHE-601, LEU-240, ALA-602, ALA-628	4	ASP-232, HIS-626
	Mk.3 ^b^	−8.7	ILE-396, TRP-467, TRP-432, MET-470, ILE-233, ALA-234, TRP-329, PHE-236, PHE-601, LEU-240, ALA-602, ALA-628	4	ASP-357, HIS-626
HMG-CoA reductase	mevastatin ^a^	−6.0	LEU-562, LEU-853, ALA-751, MET-657, VAL-683, LEU-857	2	/
	Cp.2 ^b^	−9.0	LEU-562, LEU-853, ALA-751, MET-657, VAL-683, MET-655, ALA-859, ALA-856, ALA-525	4	LYS-735
	Cp.4 ^b^	−8.2	LEU-562, LEU-853, ALA-751, MET-657, VAL-683, LEU-857, ALA-564, ALA-856, MET-655	3	ARG-590

^a^ original inhibitor; ^b^ good inhibitors from acylglycosides flavones and their metabolites; ^c^ the common hydrogen bond residues with original inhibitor.

## Supplementary Materials

Supplementary materials can be found at http://www.mdpi.com/1422-0067/20/3/494/s1. Figure S1. The detailed data of isolated compounds with high purity identified by NMR and TOF-MS was depicted in Supplementary Materials.
