# Supplementary material for: New Acylglycosides Flavones from Fuzhuan Brick Tea and Simulation Analysis of Their Bioactive Effects
Source: Int J Mol Sci. 2019 Jan 24;20(3):494. doi: 10.3390/ijms20030494 (PMC6387181; doi:10.3390/ijms20030494)

Supplemental materials

Figure S1. The detailed data of isolated compounds with high purity identified by NMR and TOF-MS depicted as followed:

Compound 1

^{13}C-NMR

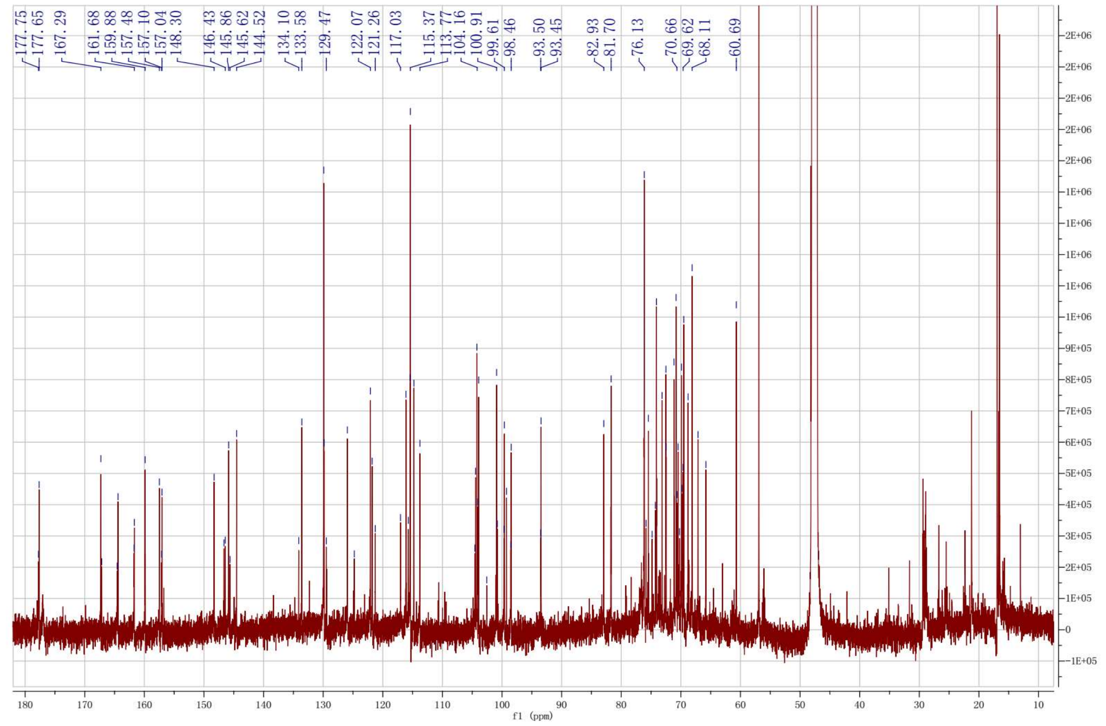

^{1}H-NMR

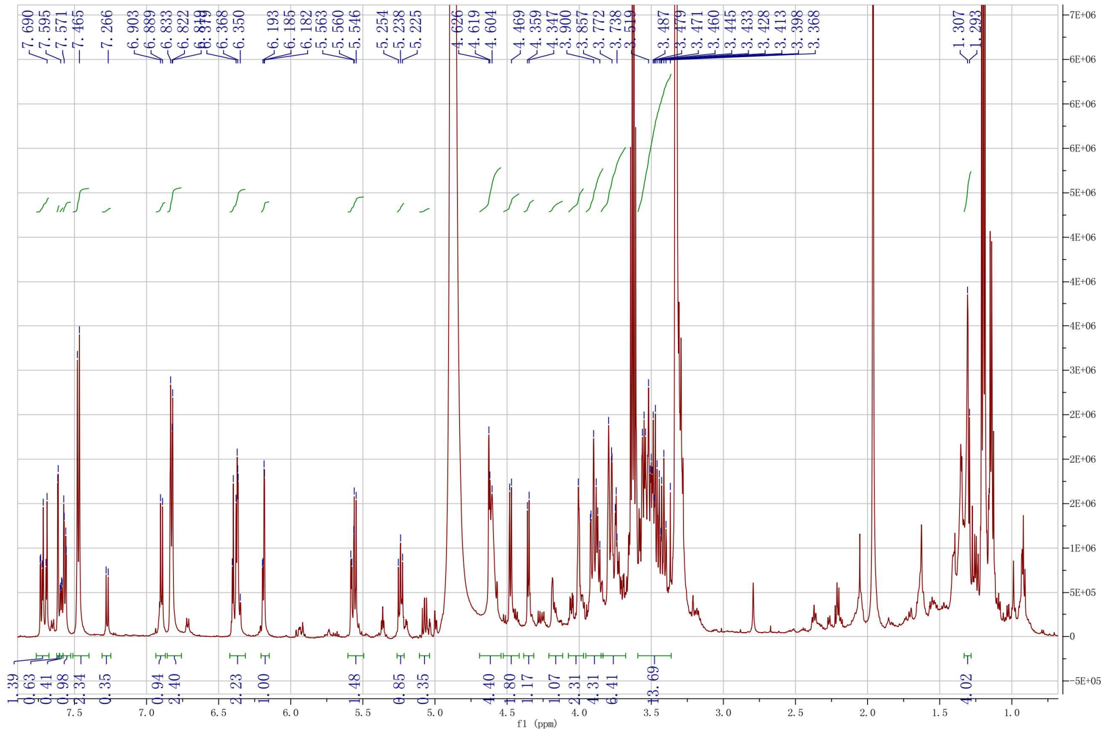

H-H COSY

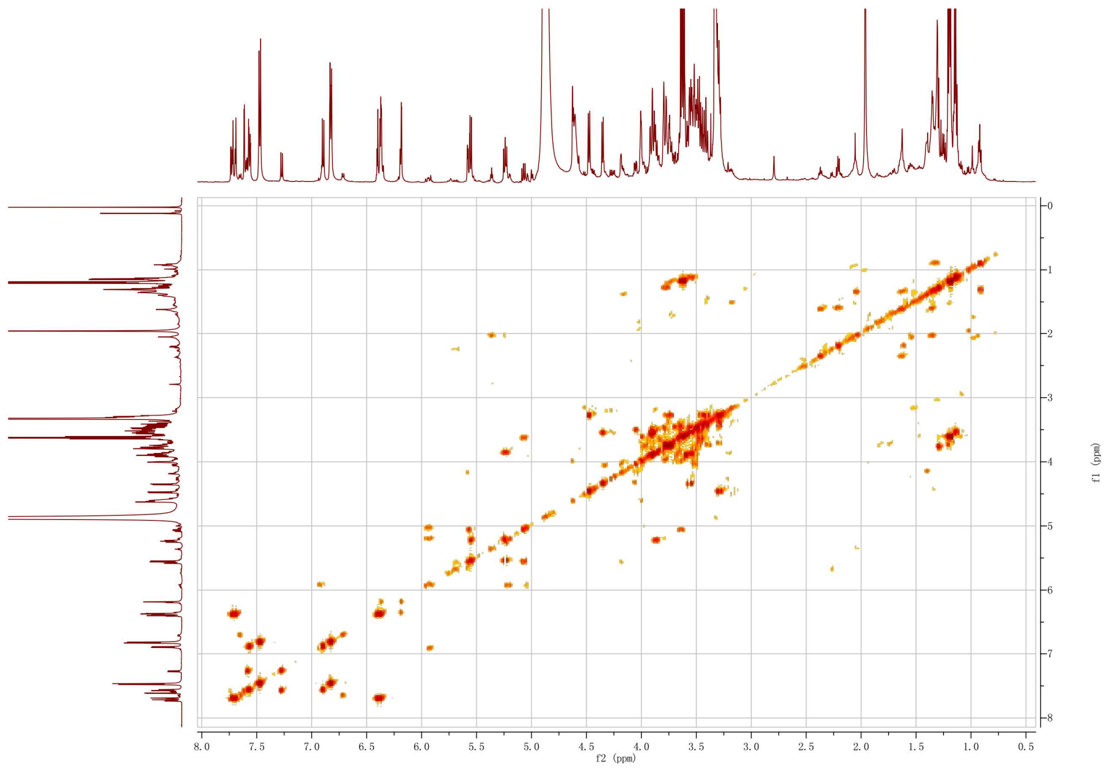

HMBC

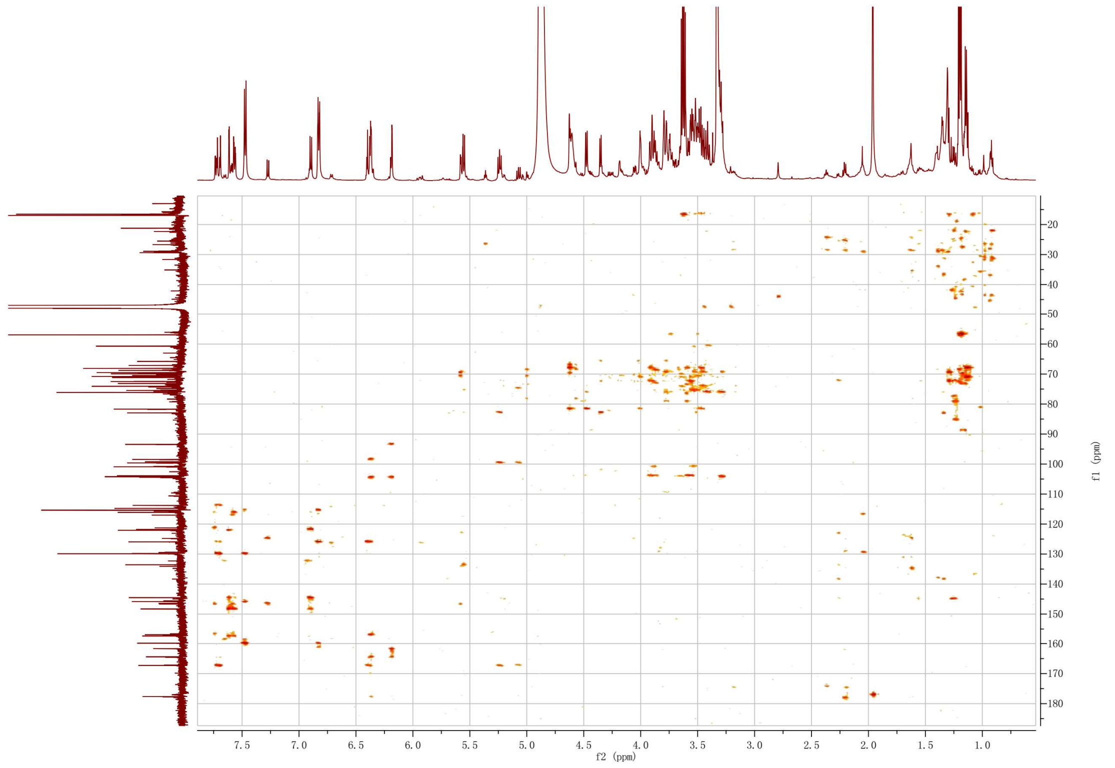

HSQC

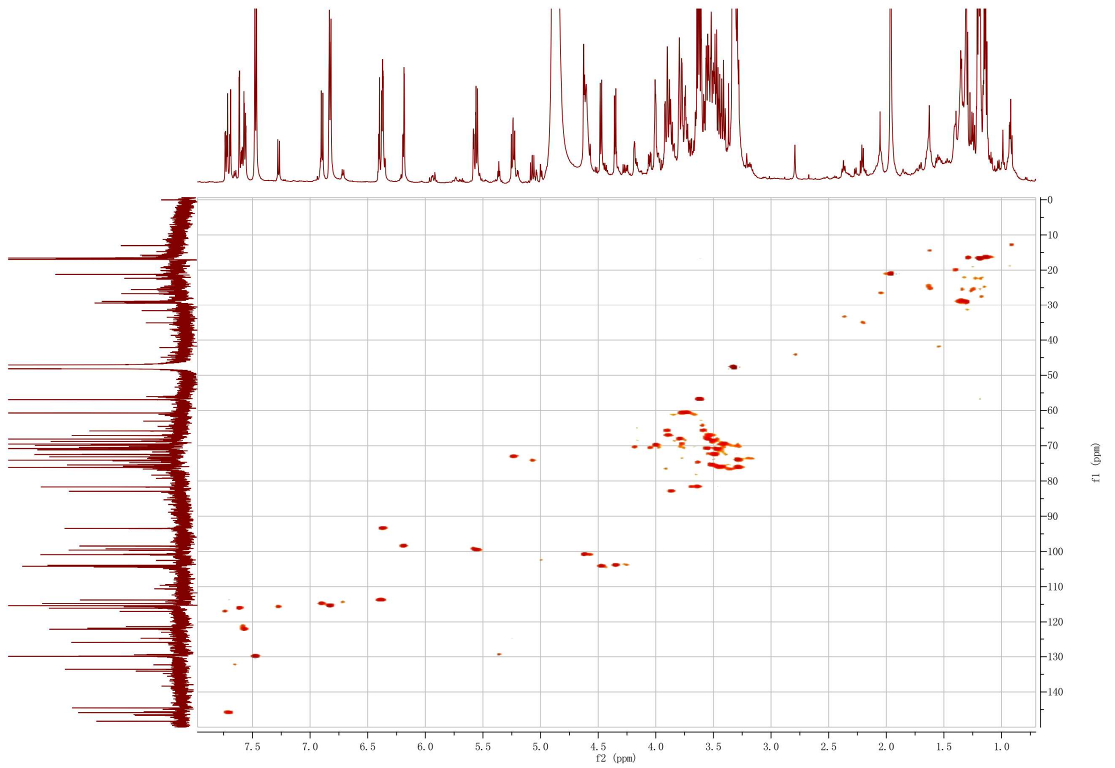
f2 (ppm)
f1 (ppm)

MS

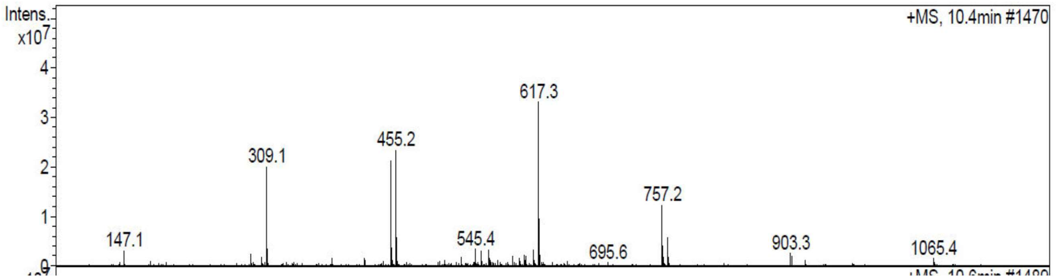
Intens.
x10⁷
+MS, 10.4min #1470
617.3
455.2
309.1
757.2
147.1
545.4
695.6
903.3
1065.4

Compound 2

^{13}C-NMR

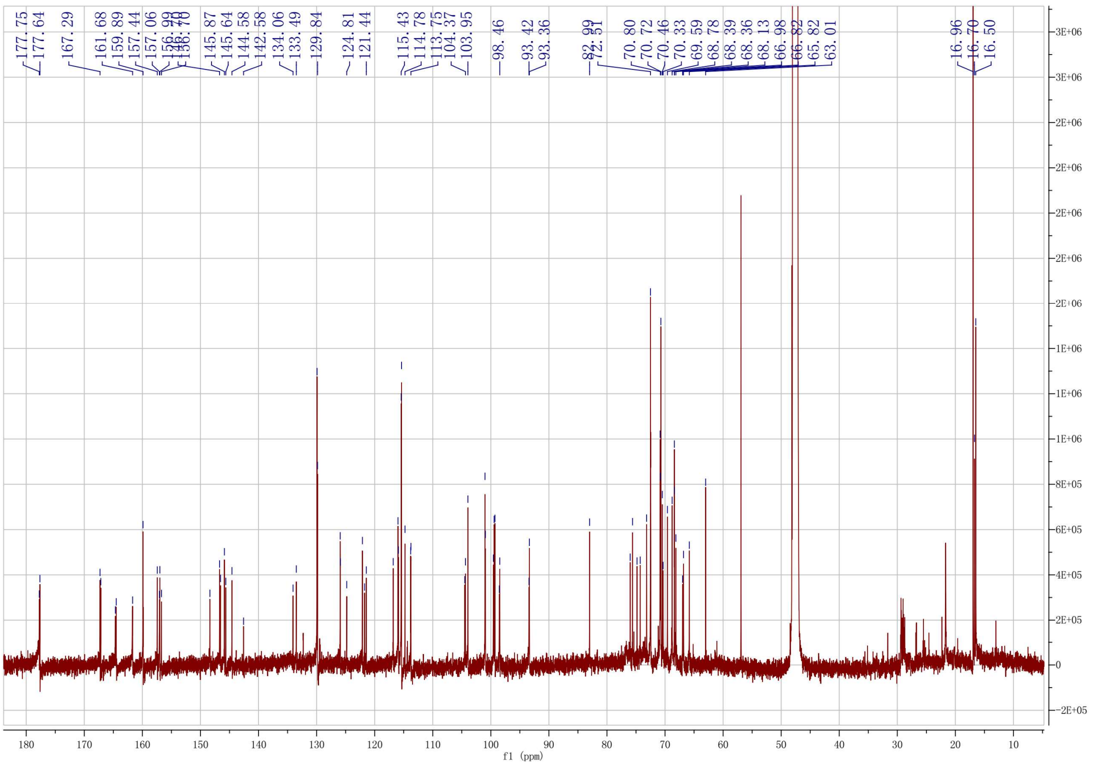

^{1}H-NMR

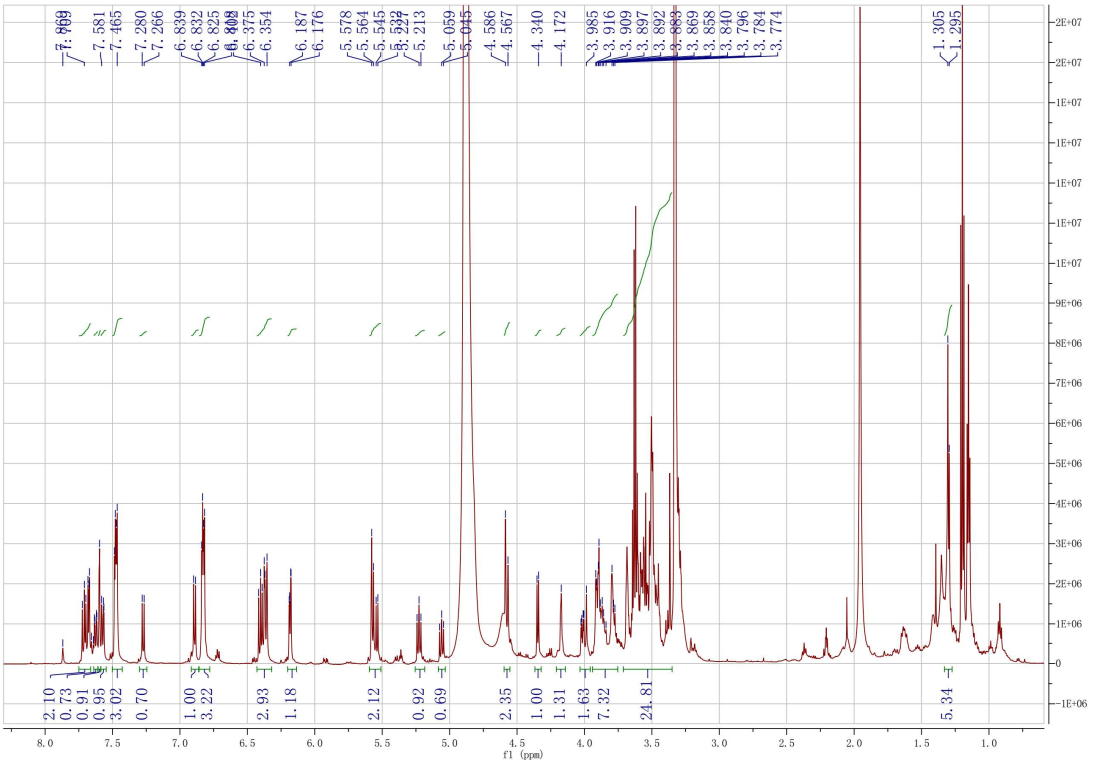

H-H COSY

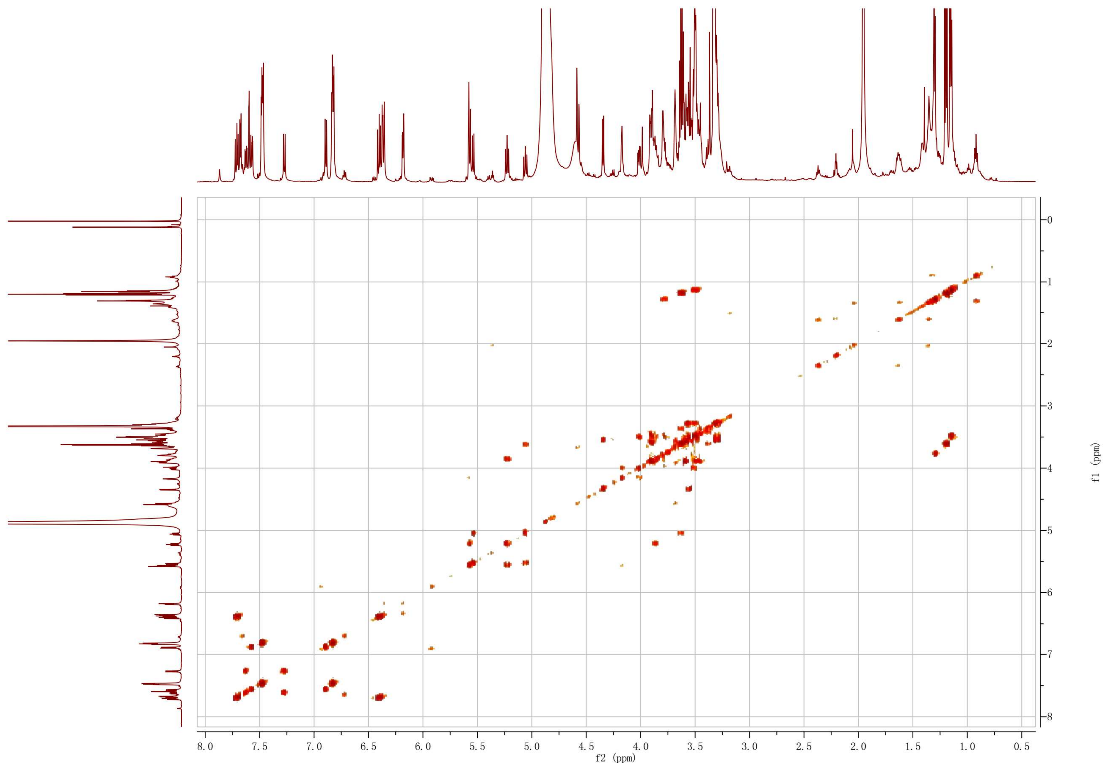

HMBC

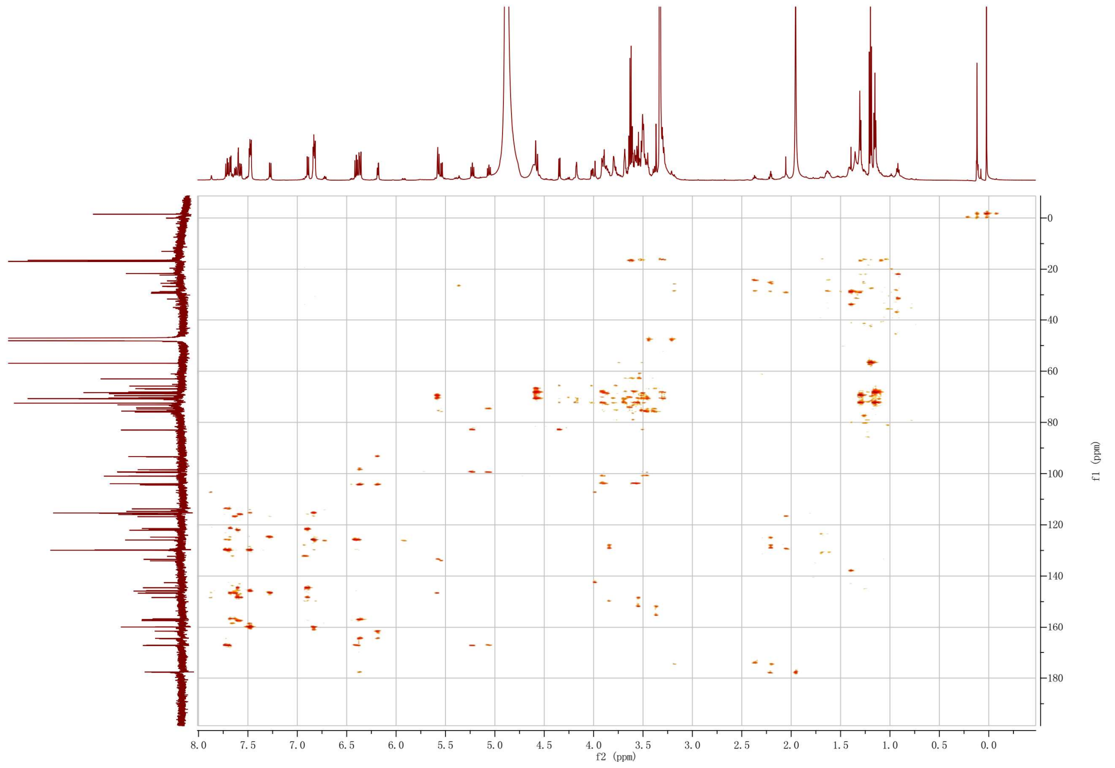

HSQC

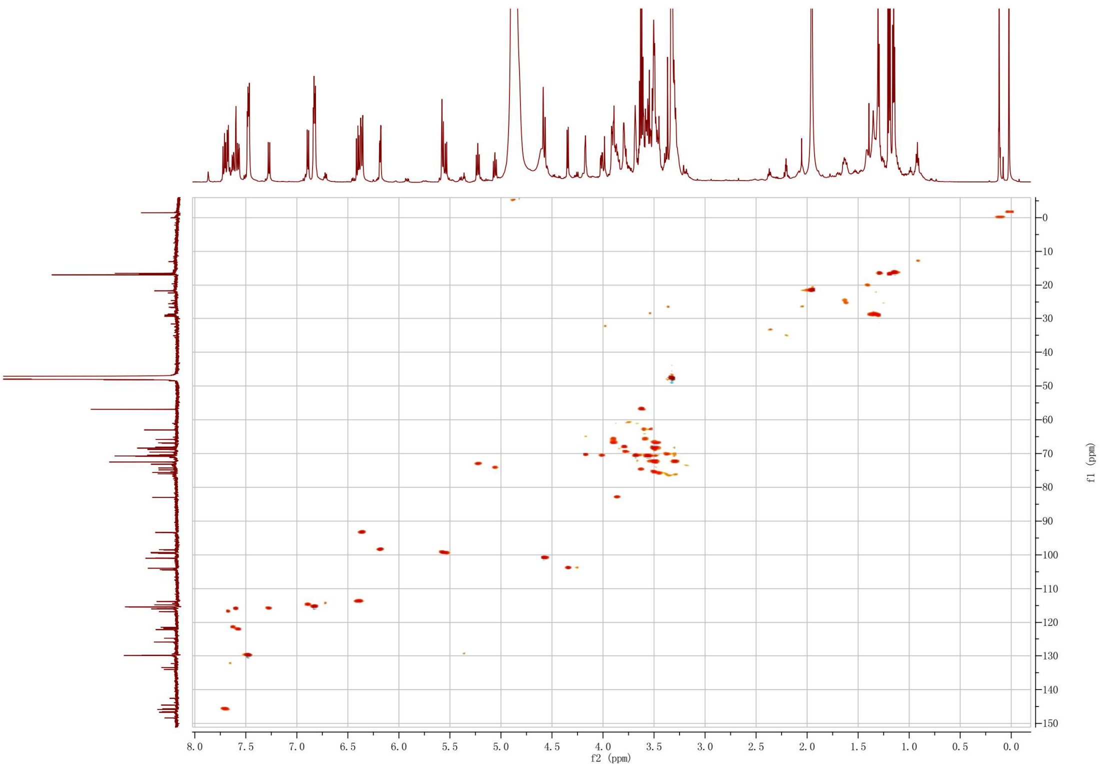
f2 (ppm)
f1 (ppm)

MS

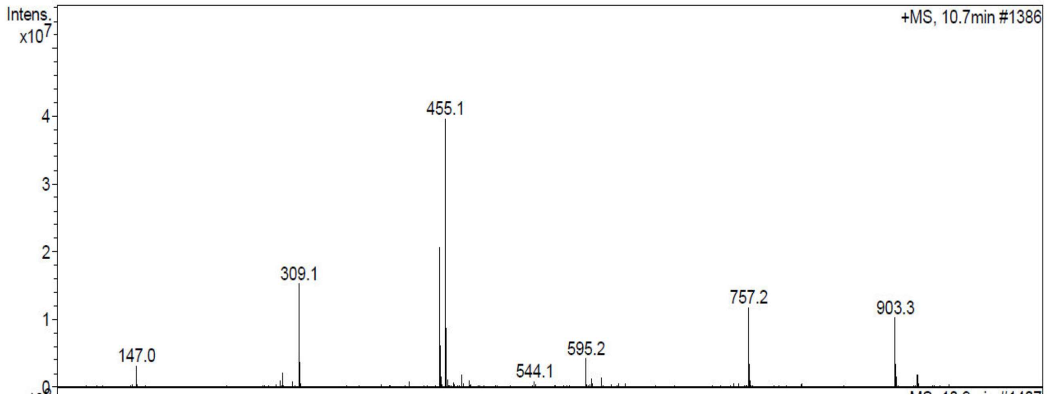
Intens.
x10^7
+MS, 10.7min #1386
455.1
309.1
147.0
544.1
595.2
757.2
903.3

Compound 3

^{13}C-NMR

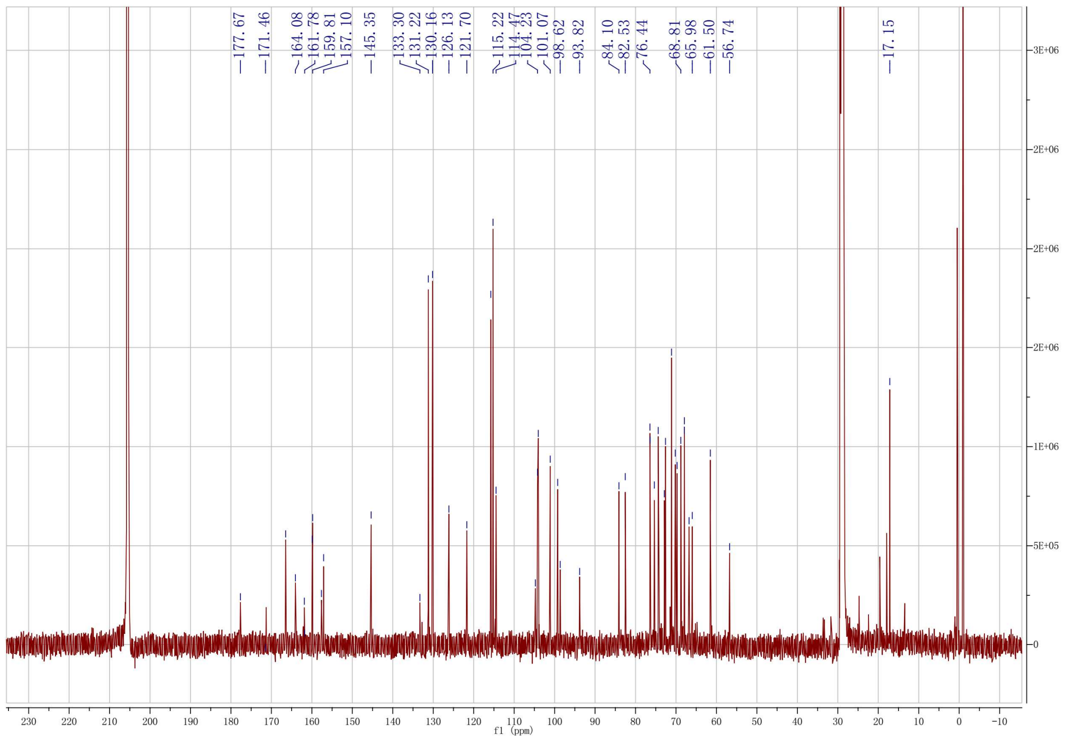

^{1}H-NMR

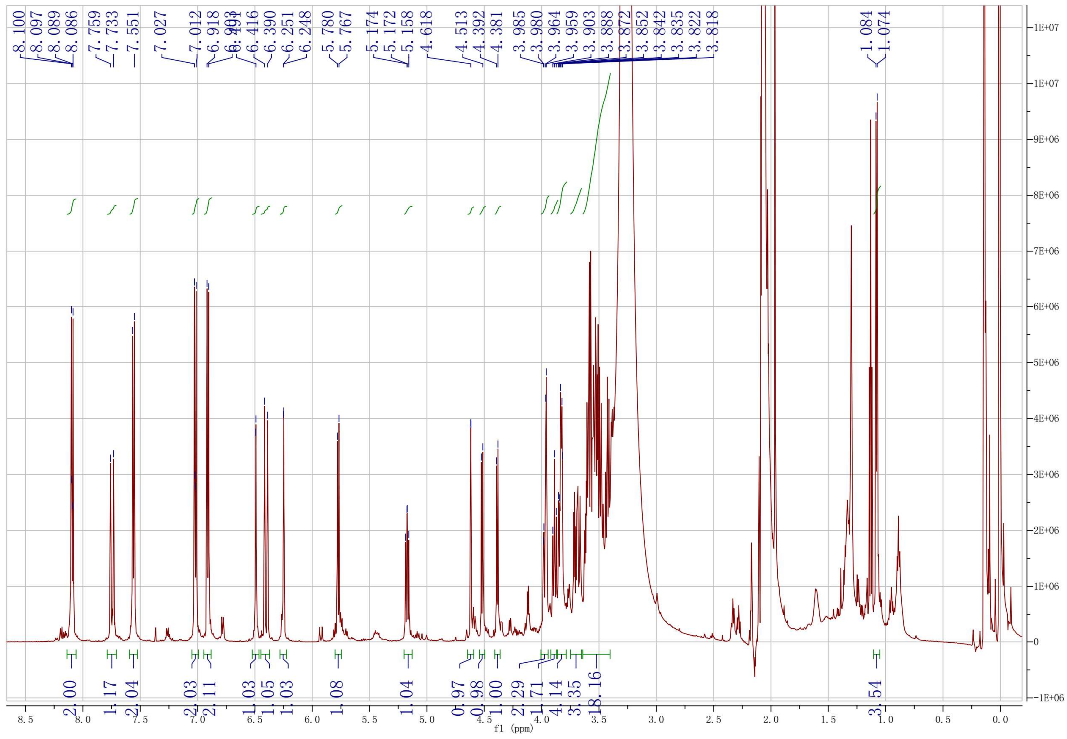

H-H COSY

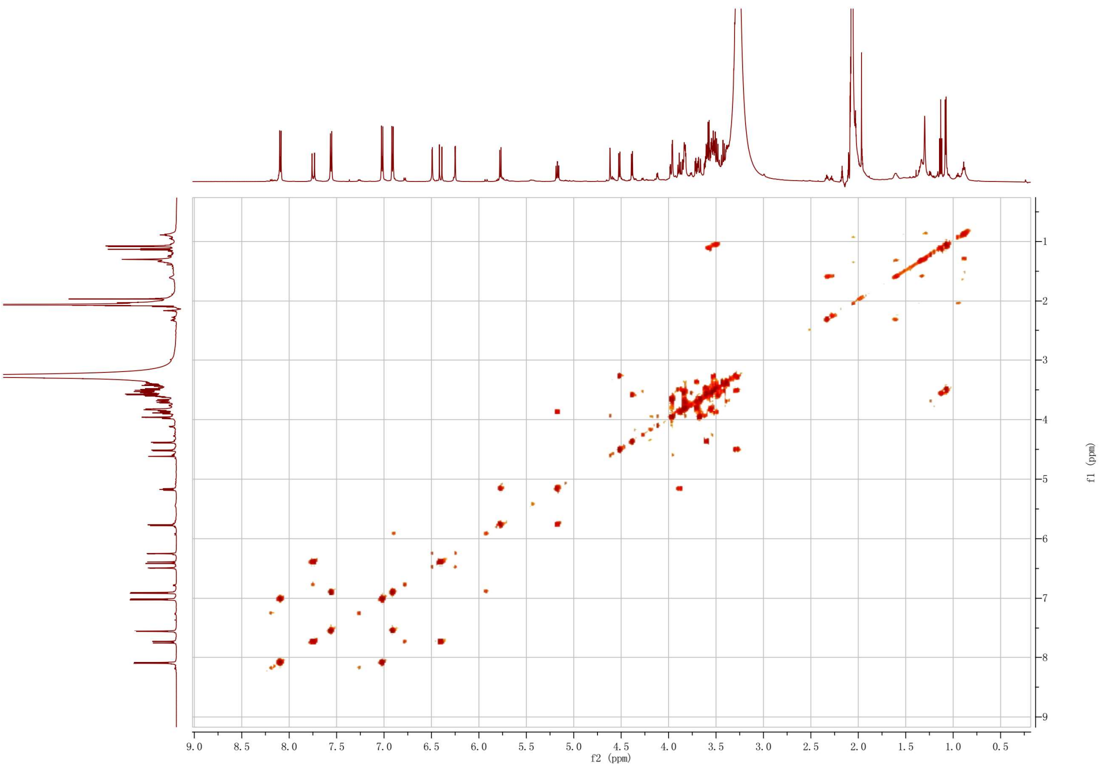

HMBC

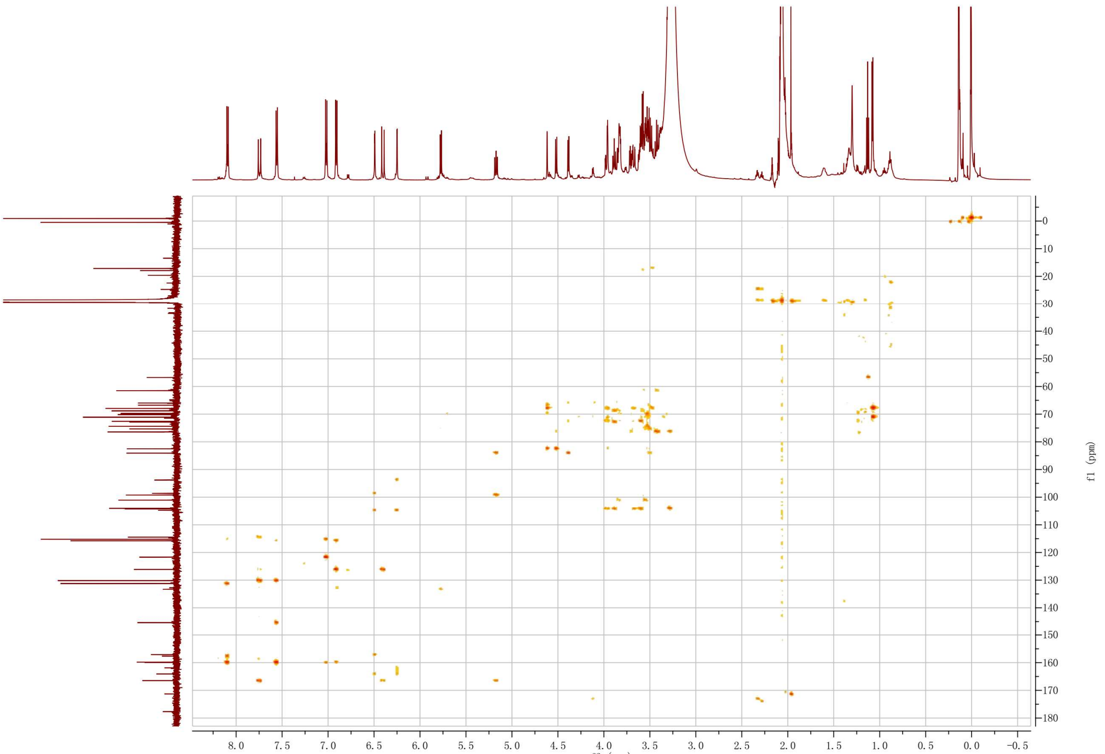

HSQC

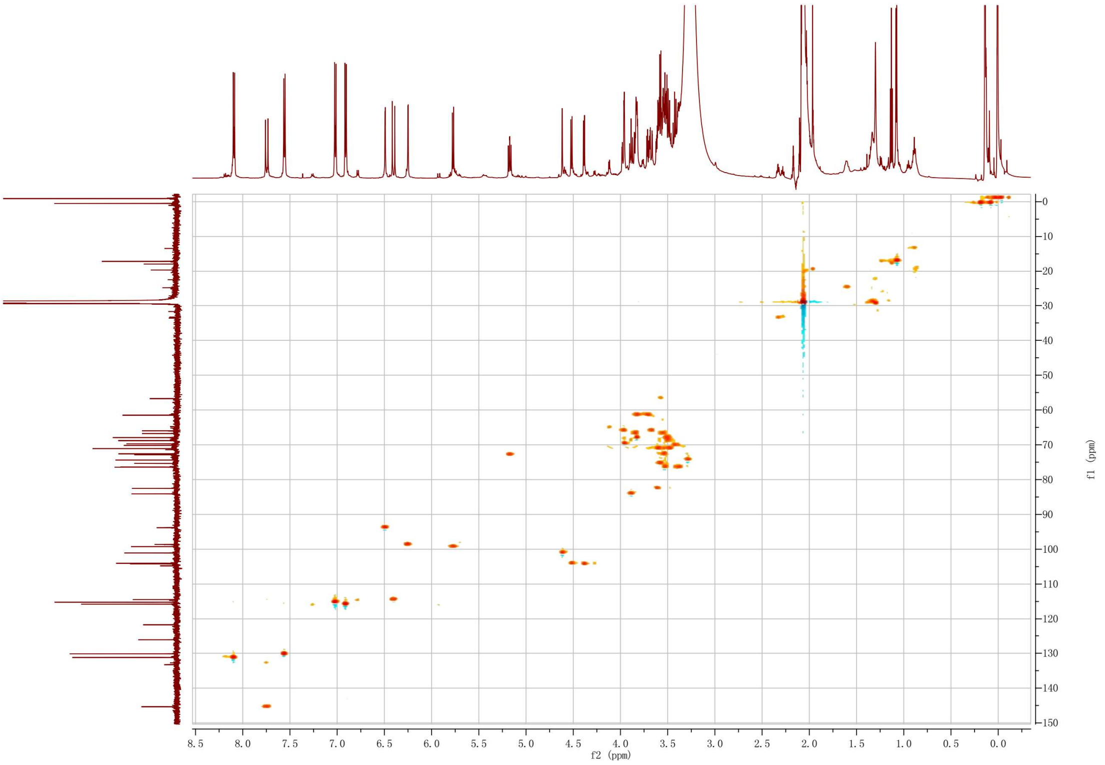
f2 (ppm)
f1 (ppm)

MS

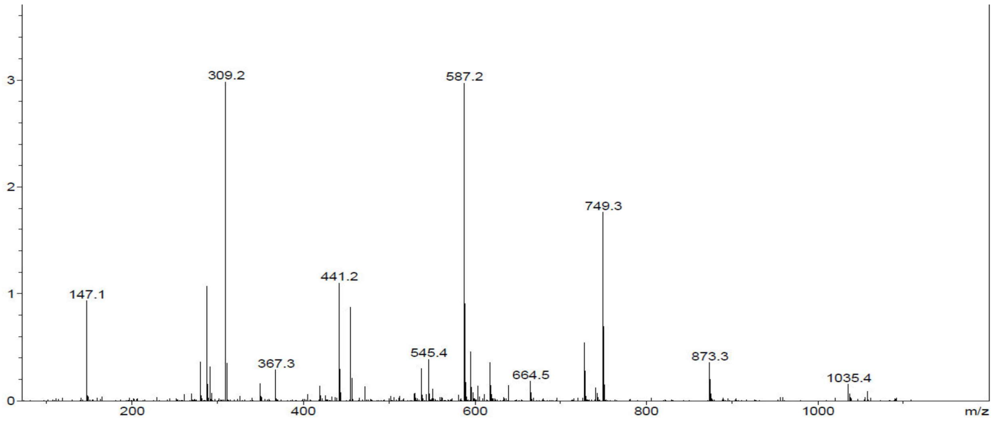
309.2
587.2
749.3
147.1
441.2
367.3
545.4
664.5
873.3
1035.4
m/z

Compound 4

^{13}C-NMR

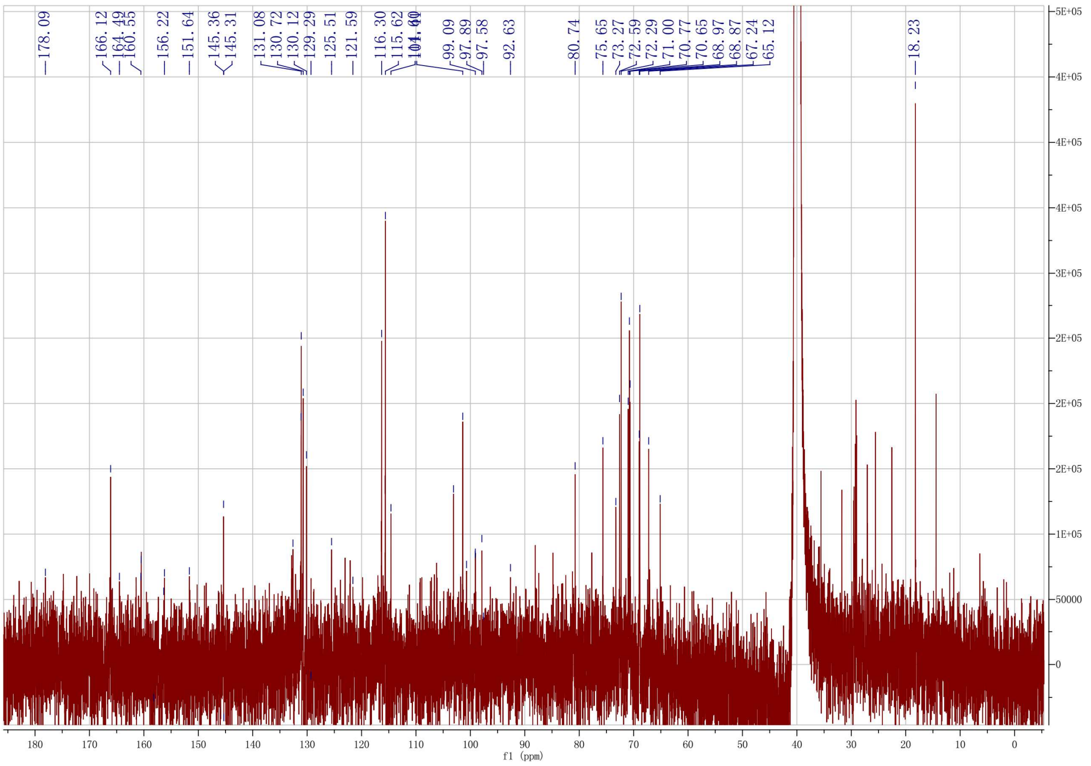

^{1}H-NMR

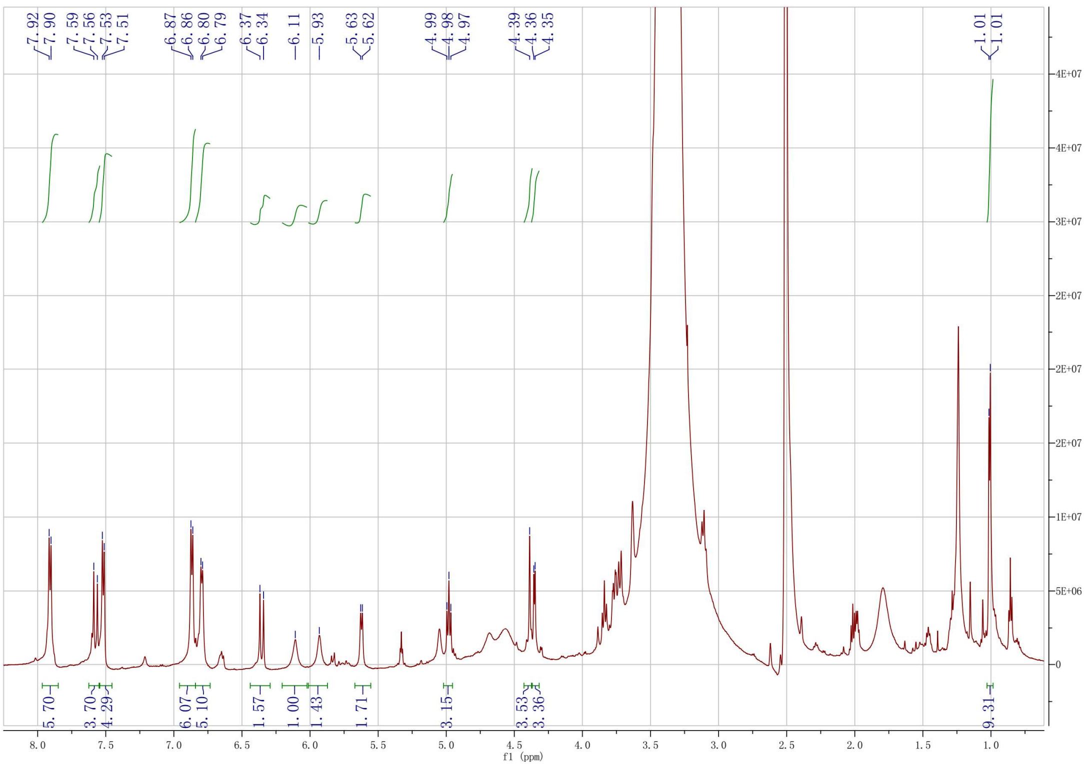

H-H COSY

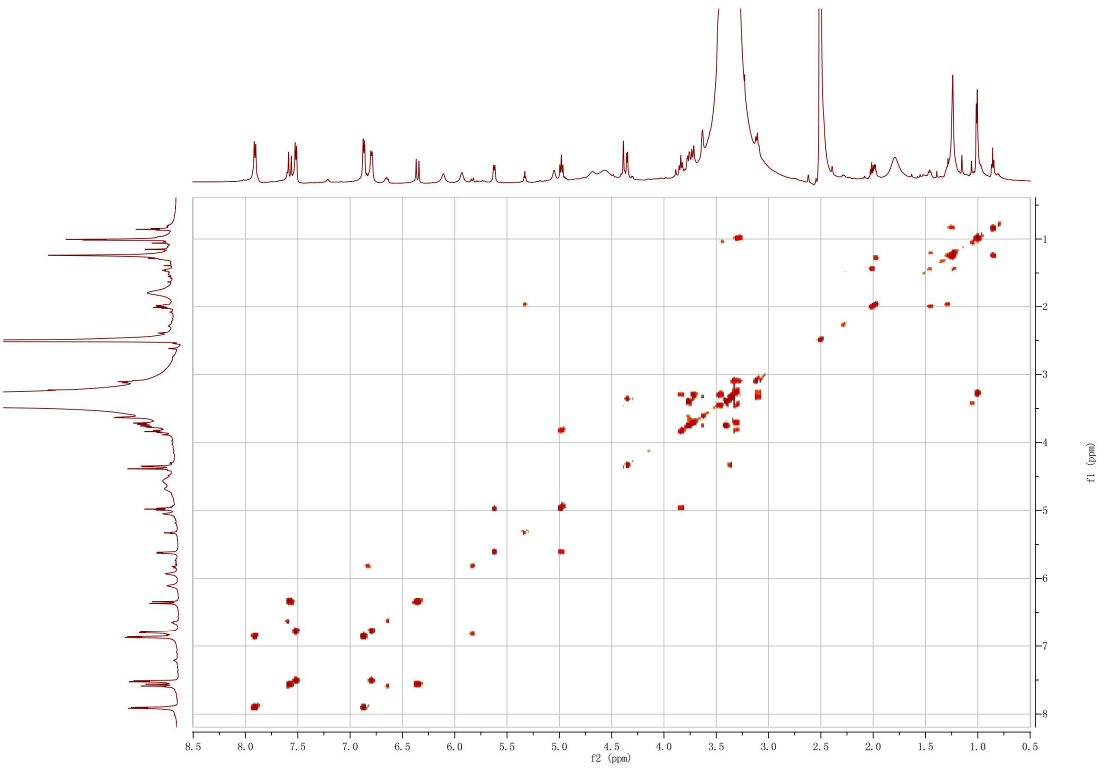

HMBC

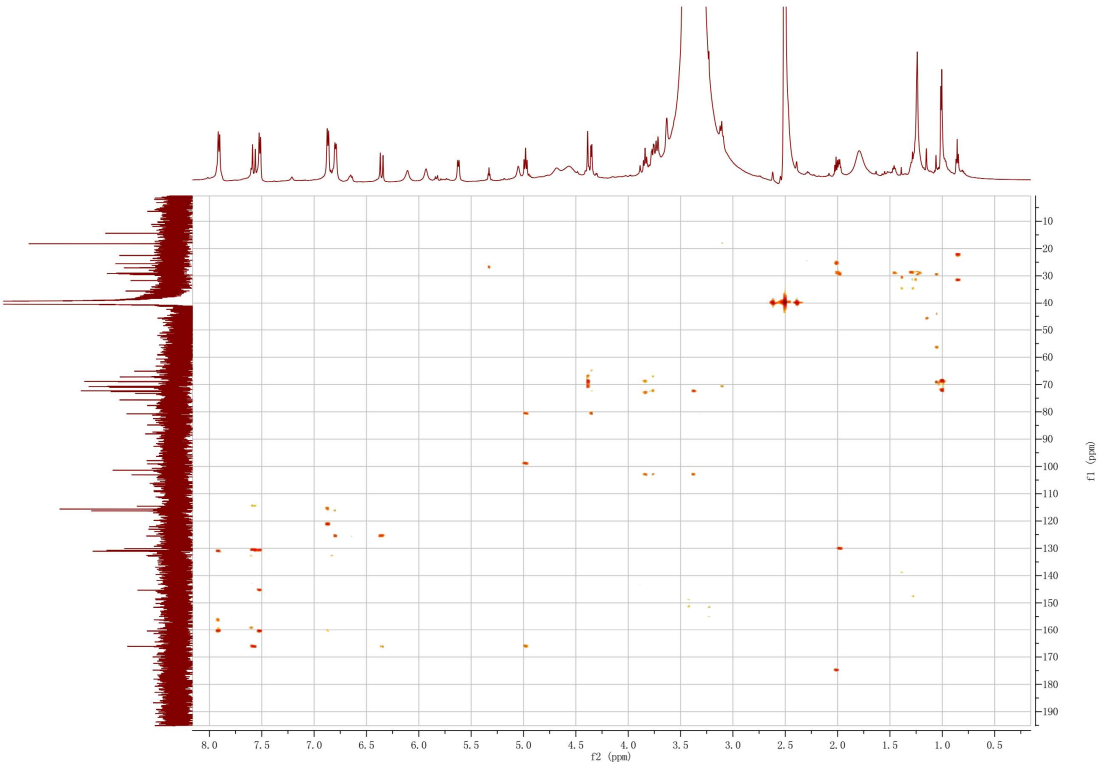

HSQC

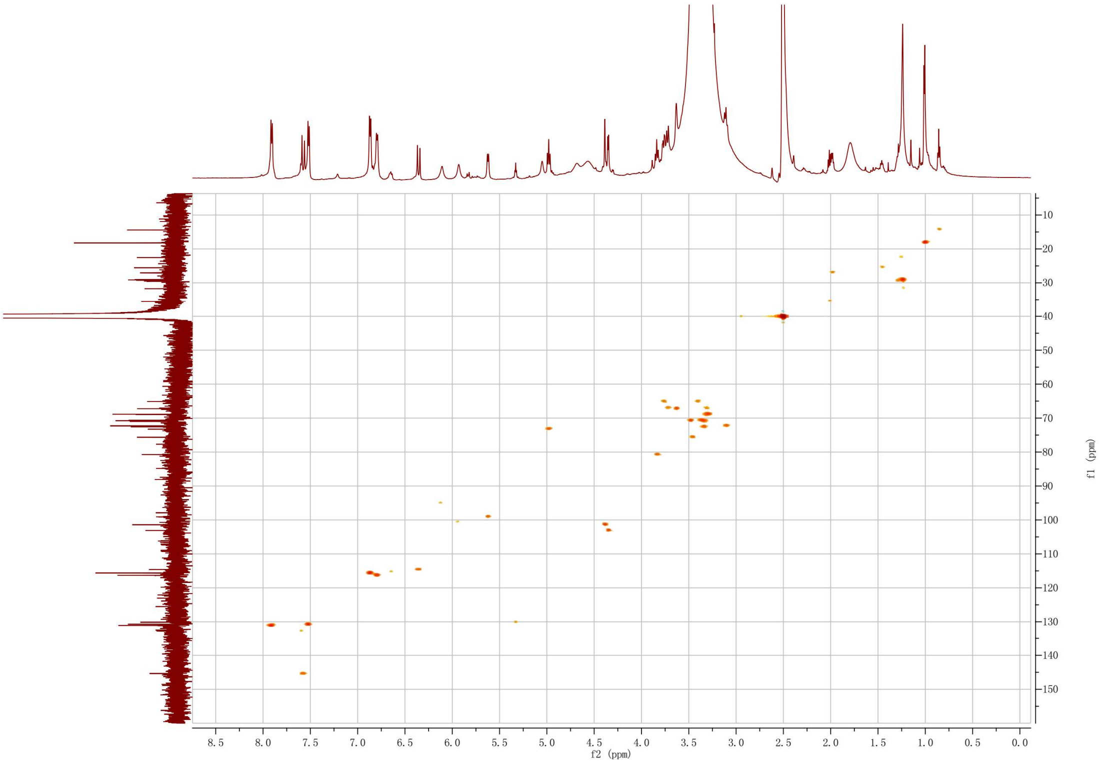

MS

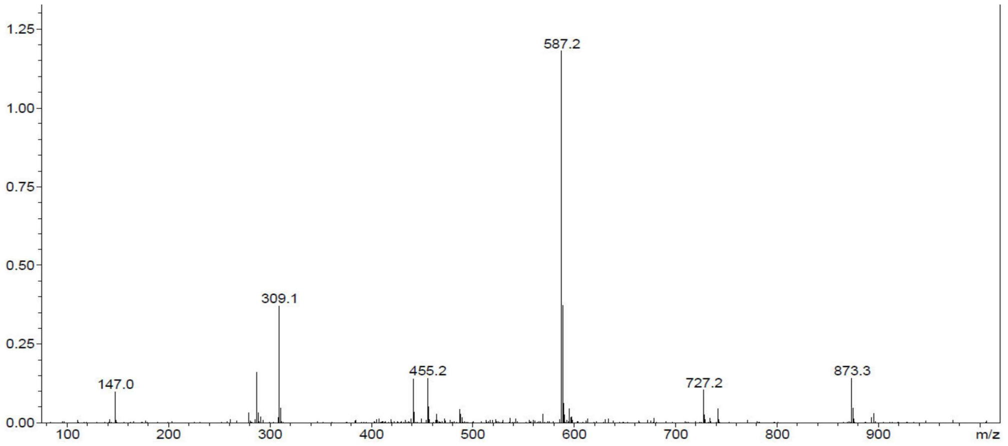

Supplement: Supplementary file 1 [file ijms-20-00494-s001.pdf]
